# Mammographic image unsharpness: a predictable phenomenon?

**DOI:** 10.1186/bcr2953

**Published:** 2011-11-04

**Authors:** A Coltart, C Blythe, M Hair

**Affiliations:** 1 South West of Scotland Breast Screening Unit, Irvine, UK; 2 Queen Margaret University, Edinburgh, UK; 3 Ayrshire and Arran Health Board, Kilmarnock, UK

## Introduction

In 2009, the South West of Scotland Breast Screening Unit (SWSBSU) had to repeat 236 examinations due to mammographic image unsharpness. Such recalls were undesirable because of radiation safety issues, anxiety caused and cost. The aim of this study was to model predictors of recall that could be used in clinical practise to help reduce the numbers of repeat examinations required due to blurring.

## Methods

This retrospective study compared two sample groups (*n *= 118), randomly selected from a cohort of women who attended the SWSBSU in 2009 (*n *= 16,194). Tests of significance were used to compare a range of variables in each group and logistic regression was employed to produce four predictor models for recall. Statistical analysis was carried out by the authors using SPSS version 18.

## Results

A 12 mm increase in compressed breast thickness (CBT), a 50 mAs increase in exposure or a 350 ms increase in exposure time will double the odds of recall due to unsharpness. Also, an increase of 2 kVp, when imaging a breast with a CBT of 60 mm, will reduce the odds of recall by approximately 39%.

## Conclusion

CBT is a major determinant of recall due to unsharpness. Within the context of the reported increase in average CBT [1] and rising national obesity, reconsideration of kVp selection criteria may be necessary to minimise the incidence of recall due to unsharpness.

## References

[B1] Robinson M Kotre C Trends in compressed breast thickness and radiation dose in breast screening mammography Br J Radiol 2008 81 214 218 10.1259/bjr/90916004 18270295

